# An Insight Into the Mechanism of Plant Organelle Genome Maintenance and Implications of Organelle Genome in Crop Improvement: An Update

**DOI:** 10.3389/fcell.2021.671698

**Published:** 2021-08-10

**Authors:** Kalyan Mahapatra, Samrat Banerjee, Sayanti De, Mehali Mitra, Pinaki Roy, Sujit Roy

**Affiliations:** Department of Botany, UGC Center for Advanced Studies, The University of Burdwan, Burdwan, India

**Keywords:** crop improvement, DNA damage response, homologous recombination, MSH1, organelle genome, retrograde signaling, SOG1, WHIRLY family proteins

## Abstract

Besides the nuclear genome, plants possess two small extra chromosomal genomes in mitochondria and chloroplast, respectively, which contribute a small fraction of the organelles’ proteome. Both mitochondrial and chloroplast DNA have originated endosymbiotically and most of their prokaryotic genes were either lost or transferred to the nuclear genome through endosymbiotic gene transfer during the course of evolution. Due to their immobile nature, plant nuclear and organellar genomes face continuous threat from diverse exogenous agents as well as some reactive by-products or intermediates released from various endogenous metabolic pathways. These factors eventually affect the overall plant growth and development and finally productivity. The detailed mechanism of DNA damage response and repair following accumulation of various forms of DNA lesions, including single and double-strand breaks (SSBs and DSBs) have been well documented for the nuclear genome and now it has been extended to the organelles also. Recently, it has been shown that both mitochondria and chloroplast possess a counterpart of most of the nuclear DNA damage repair pathways and share remarkable similarities with different damage repair proteins present in the nucleus. Among various repair pathways, homologous recombination (HR) is crucial for the repair as well as the evolution of organellar genomes. Along with the repair pathways, various other factors, such as the MSH1 and WHIRLY family proteins, WHY1, WHY2, and WHY3 are also known to be involved in maintaining low mutation rates and structural integrity of mitochondrial and chloroplast genome. SOG1, the central regulator in DNA damage response in plants, has also been found to mediate endoreduplication and cell-cycle progression through chloroplast to nucleus retrograde signaling in response to chloroplast genome instability. Various proteins associated with the maintenance of genome stability are targeted to both nuclear and organellar compartments, establishing communication between organelles as well as organelles and nucleus. Therefore, understanding the mechanism of DNA damage repair and inter compartmental crosstalk mechanism in various sub-cellular organelles following induction of DNA damage and identification of key components of such signaling cascades may eventually be translated into strategies for crop improvement under abiotic and genotoxic stress conditions. This review mainly highlights the current understanding as well as the importance of different aspects of organelle genome maintenance mechanisms in higher plants.

## Introduction

In plants, mitochondria and chloroplast are double membrane bound semi autonomous organelles having self contained genetic materials (mt-DNA and cp-DNA, respectively) and equipped with the associated molecular machinery for regulation of gene expression (Gutman and Niyogi, 2009; Smith and Keeling, 2015; Gray, 2017; Peralta-Castro et al., 2020). Both chloroplast and mitochondrial DNA have originated endosymbiotically from cyanobacteria and α-proteobacteria, respectively (Sagan, 1967; Dyall et al., 2004; Chevigny et al., 2020). The organellar DNA has also been shown to encode at least part of the genetic information required to accomplish some of the fundamental processes, such as photosynthesis and respiration (Saki and Prakash, 2017; Sakamoto and Takami, 2018; Duan et al., 2020). Plant mitochondrial DNA exhibits remarkable variation in size and predominantly linear in structure, while the characteristic features of chloroplast genome remain fairly constant across various plant species and may exist in both linear and circular forms (Morley et al., 2019; Chevigny et al., 2020). Both of the organellar genomes are present in high copy numbers. However, the copy number usually varies within different tissue types during different stages of plant development (Oldenburg and Bendich, 2015; Krupinska et al., 2020).

Due to their proximity with the reactive oxygen species (ROS) generating electron transport system, both mitochondrial and chloroplast genome are threatened by diverse forms of oxidative damages (Boesch et al., 2009). Therefore, maintenance of the stability of the organellar genome along with the nuclear genome is an absolute requirement for plants for sustaining their normal growth and development. Thus, as like the nuclear genome, faithful replication and repair of organellar genomes are also crucial to avoid genome instability, which may eventually cause potentially detrimental effects on phenotypes (Ahmad and Nielsen, 2020). To maintain genome integrity under adverse conditions, plant mitochondria and chloroplast have evolved with an extensive regulatory mechanisms to counteract the deleterious effects of DNA damage (Maréchal and Brisson, 2010; Oldenburg and Bendich, 2015; Ahmad and Nielsen, 2020). Plant mitochondria and chloroplast have been found to possess well-developed base excision repair (BER) pathways, comparable to the nuclear genome to cope up with different forms of oxidative DNA damages (Boesch et al., 2009; Peralta-Castro et al., 2020). Besides oxidative lesions, double-strand breaks (DSBs), which are also generated in the organellar genome either spontaneously or in response to other stress signals, are predominantly repaired by various homology-dependent repair pathways, as unlike the nuclear genome, both the organelles lack the non-homologous end-joining NHEJ pathways (Maréchal and Brisson, 2010).

The endosymbiotic origin of mitochondria and chloroplast from cyanobacteria and α-proteobacteria, respectively has indicated the presence of prokaryotic mode of replication and repair machineries in both the organelles (Ahmad and Nielsen, 2020). However, these replication or recombination/repair proteins, which participate in genome stability maintenance mechanisms in mitochondria and chloroplast, are encoded by either of these organellar genomes (Morley et al., 2019; Brieba, 2019). Analyses of the replication and repair machinery in mitochondria and chloroplast have revealed that the protein components involved in replication and repair machinery are mainly encoded by the nuclear genome and then subsequently targeted to the respective organelle (Saki and Prakash, 2017; Ahmad and Nielsen, 2020; Duan et al., 2020). Except for about 13 mitochondrial genome encoded proteins, more than 1500 proteins in the overall mitochondrial proteome are encoded by various nuclear genes and are targeted to mitochondria for maintenance of mitochondrial genome stability (Chacinska et al., 2009; Van Houten et al., 2016; Saki and Prakash, 2017). On the other hand, more than 95% of chloroplast proteins, including those associated with the maintenance of cp-DNA have been shown to be encoded by the nuclear genome (Green, 2011), which contains all the required genetic information for chloroplast functioning and plant survival (Woodson and Chory, 2008).

Both mitochondria and chloroplast genomes have been shown to experience considerable magnitude of homologous recombination and gene conversion events between comparable DNA sequences (Maréchal and Brisson, 2010; Wu et al., 2020). Chloroplast genome contains large inverted repeats, which generally exhibit slower sequence evolution as compared to single-copy regions, suggesting improved precision of error correction in the presence of easily available homologous templates (Wolfe et al., 1987; Zhu et al., 2016). Various DNA repair mechanisms in the organelles have been found to play important roles in protecting their genome stability and showed close association regarding their evolution. However, despite the highly oxidative environment, the rate of sequence evolution in both chloroplast and mitochondrial genome has been much slower as compared to nuclear genome. The comparatively slower rate of evolution of plant mt-DNA and cp-DNA with reduced substitution rate might be due to the activity highly efficient homologous recombination pathway, which mainly corrects the lesions by gene conversion (Wolfe et al., 1987; Gualberto and Newton, 2017; Chevigny et al., 2020). Recent studies have revealed that members of *mutS* mismatch repair family protein MSH1, unique in plants, is dually targeted to both chloroplast and mitochondria and play crucial role for maintaining the slow rate of mutation in the organellar genome (Virdi et al., 2016; Wu et al., 2020). Disrupting the function of this gene has been found to increase the frequency of mitochondrial and chloroplast sequence variants approximately 10-fold to 1,000-fold in the model plant *Arabidopsis thaliana* (Wu et al., 2020).

As both mitochondria and chloroplast are believed to have originated from the engulfment of bacterium by eukaryotic cell and most genes were either lost or transferred to the nucleus after the endosymbiosis through endosymbiotic gene transfer (Timmis et al., 2004), the proper coordination of nuclear and organellar genome is therefore essential for ensuring the stability of both the organelles as well as retaining the cellular integrity (Kobayashi et al., 2009; Duan et al., 2020). Studies on genome communication mechanisms, including anterograde (nucleus to organelle) and retrograde (organelle to the nucleus) signaling has been primarily focused regarding the coordination of gene expression between nuclear and organellar genomes (Woodson and Chory, 2008; Chan et al., 2016). Interestingly, the activation and suppression of DNA damage response or DDR in plant organelles depends on the transportation of different proteins from the nucleus to the organelles. Organellar double-strand breaks are shown to be processed by different organellar single-strand binding proteins (OSBs) or the WHIRLY family proteins (Cappadocia et al., 2010). These nuclear genome encoded proteins are either mitochondrial-targeted (OSB1, OSB4, WHY2), chloroplast targeted (OSB2, WHY1, WHY3), or targeted to both the organelles (OSB3) through the anterograde signaling pathway (Krause et al., 2005; Mare’chal et al., 2008, 2009). The loss of function of *WHIRLY* family genes leads to disruption of homologous recombination repair and reduction in recombination events within sequences exhibiting microhomology (Cappadocia et al., 2010). In addition, some recent studies have demonstrated that chloroplast genome instability also affects the status of the nuclear genome, as the SOG1 transcription factor (the plant homolog of mammalian tumor suppressor p53), which acts as the central regulator of plants’ DNA damage responses, has been shown to promote endoreduplication and modulate cell-cycle progression from chloroplast to nucleus through retrograde signaling in response to chloroplast genome instability (Duan et al., 2020). Therefore, maintenance of mitochondrial and chloroplast genomes and the transcription of genes encoded by the organellar genomes are primarily under the control of the nucleus.

The homologous recombination (HR) based repair is considered as the most error free mechanism of DSB repair and represent one of the essential source of genetic diversity and new allelic combination in plants. Therefore, understanding the HR pathway and its interactions with other repair pathways is essential for manipulation of HR events as part of the crop improvement program. The presence of highly efficient homology-dependent DSB repair in the organellar genome has opened up new possibilities in the area of genome transformation in the context of the overall goal of crop improvement (Li et al., 2021). Along with highly efficient homologous recombination system for precise transgene insertion, other characteristics, including absence of epigenetic regulations (such as gene silencing and positional effects) with the potential for expressing foreign proteins at a considerably higher level (approximately 70%) and increased biosafety due to the exclusion of chloroplasts from pollen transmission, chloroplast genome manipulation thus offers attractive alternative over nuclear genome for transgene integration (Oey et al., 2009; Bock, 2015; Boehm and Bock, 2019; Li et al., 2021). Recent studies have demonstrated highly efficient chloroplast transformation in *Arabidopsis thaliana* (Ruf and Bock, 2011). Furthermore, striking similarities between chloroplast and mitochondrial genome have also indicated the possibility of mitochondrial transformation by designing suitable strategies. However, successful mitochondrial transformation in higher plants has not been reported so far. Based on this information, this review mainly summarizes the current understanding of different aspects of organelle genome maintenance mechanisms in higher plants along with the possible applications of genetic manipulations of organellar genomes in crop improvement.

## Plant Organellar Genome Architecture

Plant mitochondria and chloroplast genomes are typically represented by small circular molecules of DNA. These organellar genomes appear to share very similar structural organization with prokaryotic genomes and the mode of replication. Conversely, unlike prokaryotes, these organelles have been found to possess multiple copies of their genome (Clark et al., 2019). However, organelle genomes do not contain enough genes to survive by themselves and thus rely mainly on the nuclear genome encoded proteins.

**FIGURE 1:**
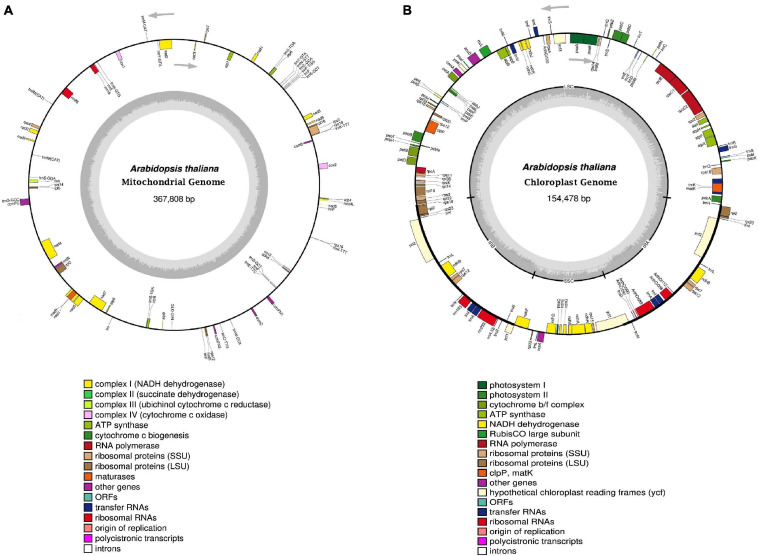
Structural organization of organelle genomes in *Arabidopsis thaliana*. **(A)** and **(B)** Circular gene map of mitochondrial and chloroplast genome, respectively. The gray arrowheads indicate the direction of genes. Genes shown inside and outside the outer circle are transcribed clockwise and anticlockwise respectively. Genes belonging to different groups are color coded. The genome coordinate and GC content are shown in the inner circle. The darker gray region corresponds to GC content, whereas the lighter gray region corresponds to AT content. In the chloroplast genome, the thick lines indicate inverted repeats (IRA and IRB), which separate the genome into small (SSC) and large (LSC) single copy regions. Genome diagrams are adapted from the output of OGDRAW (Greiner et al., 2019).

Mitochondrial and chloroplast genomes vary greatly in size and architecture both within and between eukaryotic organisms. The structure of mitochondrial genome in most animal cells is very similar, generally consisting of a circular DNA molecule of 14-20 kb (Kolesnikov and Gerasimov, 2012). It usually codes for two ribosomal RNAs, 13 polypeptides, and up to 25 tRNAs (Gualberto et al., 2014). In comparison to animals, plants possess mitochondrial genomes that are large, more complex in structure (Figure 1A) and in general the genome size ranges between 200-2000 kb (Gualberto et al., 2014). The relatively large size of the genome is mainly due to the additional amount of DNA found in large introns, non-coding regions, and repeated sequences (organized either in direct or indirect orientation) dispersed throughout the genome (Morley et al., 2019). The main difference between animal and plant mitochondrial DNA lies in the fact that in plants, it mostly exists as a collection of linear DNAs combined with smaller circular and branched molecules. Previous studies have shown that plant mitochondrial genome undergoes extensive high-frequency homologous recombination events between large repeats, resulting in multiple configurations of the genome. Plant mitochondria generally lack nuclei, but the DNA is packed into nucleoprotein particles, called nucleoids (Kucej and Butow, 2007). These are membrane-anchored particles associated with DNA and influence transcriptional regulation of mt-DNA. Mitochondrial nucleoids encompass a set of core proteins involved in the maintenance of DNA and some peripheral components of signaling pathways (Gilkerson et al., 2013). Commonly, both plant and animal mitochondrial genomes include genes encoding nine subunits (nad1, nad2, nad3, nad4, nad4L, nad5, nad6, nad7, and nad9) of the respiratory-chain complex-I, NADH dehydrogenase; one subunit of complex III, cytochrome bc_1_ (cob); three subunits of complex IV, cytochrome oxidase (cox1, cox2, cox3); and two subunits of complex V, ATP-synthase subunits (atp6, atp8), respectively. The mitochondrial genome also includes genes for ribosomal RNAs and tRNAs. Apart from these genes, plant mitochondrial genome also includes genes encoding proteins for cytochrome c biogenesis (*ccm*) and an mtt-B-like transporter (Cupp and Nielsen, 2014). The replication process of mt-DNA is much more complex in plants than in animals. Due to the large size and complexity of mitochondrial genomes, the exact mechanism for replication of mt-DNA remains unclear. However, accumulating evidences have indicated that plants use multiple strategies, like recombination-dependent replication (RDR), rolling circle mechanism similar to bacteriophage T4 DNA replication, and also the conventional bidirectional replication mechanism to replicate their mt-DNA (Gualberto et al., 2014; Morley et al., 2019).

Besides mitochondria, chloroplasts present in plant cells also contain their own genome and gene expression system. The chloroplast genome in higher plants possesses a highly conserved structural organization. In general, chloroplast genome exists in the circular form of DNA, comprising of a quadripartite structure (Figure 1B). The chloroplast genome consists of two large inverted repeats separated by a large single-copy region (LSC) and a small single-copy region (SSC), respectively (Wang and Lanfear, 2019). The chloroplast genome associated inverted repeats (IRs) are highly conserved and their length generally ranges between 20000–25000 bp (Morley et al., 2019). Genes encoded by the chloroplast genome play crucial role in photosynthesis and carbon fixation and also serve as valuable resources for molecular identification in phylogenetic studies because of their compact size, less recombination, maternal inheritance, high copy number, and moderate substitution rates, respectively (Wang W. et al., 2014; Xie et al., 2018). Generally, the chloroplast genome in angiosperms encodes about 110-130 genes and the overall size of the genome ranges between 120-180 kb, respectively (Lin et al., 2018). Initially, the chloroplast genome was thought to form only a circular structure, but recent advances have revealed that multi-branched linear structures may also exist in many angiosperms (Mower and Vickrey, 2018). The chloroplast genome typically possesses four copies of rRNA genes, several tRNA genes, and some other protein-coding genes such as ribosomal proteins, thylakoid proteins, and the large Rubisco subunit (Palmer, 1985). However, most of the chloroplast proteins are nuclear-encoded. In majority of photosynthetic organisms, chloroplast DNA is packed into nucleoids. On an average, plant, the cell possesses nearly 1000-1700 copies of chloroplast nucleoids (Dobrogojski et al., 2020). The chloroplast genome has been shown to possess approximately 45 genes, which encode the proteins involved in photosynthesis. These genes mainly include subunits of cytochrome b6f complex (6 genes), photosystem I (5 genes), photosystem II (15 genes), ATPase of chloroplast (6 genes), NADH dehydrogenase (12 genes), and Rubisco large subunit (1 gene), respectively (Dobrogojski et al., 2020). *Arabidopsis* chloroplast genome also consists of 29 genes, including 4 genes for chloroplast RNA polymerase subunits and 25 genes for components of the ribosome (Sato et al., 1999). Chloroplasts have been shown to utilize a double displacement loop strategy to initiate their DNA replication (Kunnimalaiyaan and Nielsen, 1997). In addition, the rolling circle and recombinant-dependent replication (RDR) process have also been proposed for cp-DNA replication. Several studies have revealed that just like mitochondria, chloroplasts also possess two organellar DNA polymerases, Pol1A and Pol1B, resembling bacterial DNA Pol1 (Moriyama and Sato, 2014).

### Organelle Genome Faces High Level of Oxidative Damage Due to Proximity With the ROS Generating Electron Transport Machinery

Plant mitochondria and chloroplast possess a complex network of enzyme systems for the transport of electrons associated with respiration and photosynthesis, respectively. Various ROS are generated continuously as metabolic by-products from both these organelles (Taylor and Millar, 2007; Kim, 2020). ROS are well-acknowledged for their benefits as well as deleterious effects. When present at optimum or moderate concentrations, ROS have been found to act as second messengers, thus regulating different cellular pathways. On the other hand, at high concentrations, ROS can cause substantial damage to various biomolecules, including nucleic acids and other cellular biomolecules (Sharma et al., 2012).

Since mitochondria represent one of the predominant cellular sources of ROS production, oxidative damages are by far the most studied mechanism affecting the mt-DNA stability (Meagher and Lightowlers, 2014). Different types of reactive oxygen species, including superoxide anion, hydrogen peroxide, hydroxyl radical, and singlet oxygen molecules are generated continuously as a result of electron leakage from the several sites of mitochondrial electron transport chain (ETC) (Andreyev et al., 2005; Sharma et al., 2012; Alexeyev et al., 2013). Among various ROS, O_2_^–^ is generated from direct reduction of oxygen in the flavoprotein region of NADH dehydrogenase complex, also known as complex I of the respiratory electron transport chain (Arora et al., 2002). Besides complex I, complex III or ubiquinone-cytochrome complex of the ETC is also involved in producing O_2_^–^ from oxygen (Murphy, 2009). Under normal growth condition, functioning of ETC and ATP synthesis are tightly regulated, while under various abiotic stress conditions, components of ETC are modulated, resulting in excessive reduction of various electron carriers, ending up with overproduction of ROS (Noctor et al., 2007; Blokhina and Fagerstedt, 2010). Besides mitochondria, as a light-harvesting organelle, chloroplast has also been found to inevitably produce considerable level of ROS primarily through the Photosystems (PSI and PSII). Like mitochondria, several sites of ROS production are present in chloroplast. Electron transport system connecting PSI and PSII appeared to be the main source of ROS production in the chloroplast. Under stress conditions, decreased supply of electron acceptor (NADP) leads to leakage of electrons from ferredoxin, thus resulting in the generation of O_2_^–^ from oxygen (Elstner, 1991). Leakage of the electron can also occur from 2Fe-2S and 4Fe-4S clusters, present in electron transport systems associated with PSI. In addition, electrons may also be released from the acceptor side of ETC containing Q_A_ and Q_B_ in PSII. All these contribute to the production of O_2_^–^ from oxygen (Cleland and Grace, 1999).

Both mitochondria and chloroplast possess various antioxidant compounds as the preliminary line of defense, which protect the organelles from ROS-mediated DNA damage. The ROS molecules, which have been found to escape the antioxidant mediated detoxification, may induce DNA damage through the generation of various modified bases, including thymine glycol, 8-hydroxyguanine, 5,6-dihydroxycytosine, 2,6-diamino-4-hydroxy-5-formamidopyrimidine, respectively and also some sugar break down products like 2-deoxypentose-4-ulose, erythrose and 2-deoxypentonic acid lactone, respectively. All these oxidative modifications of the bases may generate base-free sites, eventually leading to DNA strand breaks, including DSBs (Dizdaroglu et al., 2002; Evans et al., 2004; Boesch et al., 2011).

## DNA Damage Repair Mechanisms in Plant Mitochondria and Chloroplast

Maintenance of organelle genome stability is crucial for their proper functioning as well as for overall plant growth and development. Initially, it was thought that plant organelles do not possess any dedicated machinery for repairing DNA damages, and a high copy number of mitochondrial and chloroplast genome probably restrict the deleterious effects of DNA damage (Chevigny et al., 2020). In animal system, although damaged mt-DNA copies have been found to be completely degraded, similar mechanism has not yet been detected in plant organelles (Zhao, 2019; Chevigny et al., 2020). However, it is now well established that plant organelles possess their own DNA repair machinery, while not all repair the pathways have been demonstrated to date. Various components of organellar genome maintenance machinery were probably inherited from the bacterial ancestors. However, plant organelles are still highly dependent on nuclear genome encoded damage repair proteins for repairing single-strand and double-strand breaks. On the other hand, to counteract the continuous threat from various forms of oxidative damages in the organellar genome, which mainly originate because of their intrinsic association with the electron transport chain, both mitochondria and chloroplast genomes have been found to develop efficient BER pathway as like the nuclear genome. BER pathway is the first organellar repair pathway described in plants (Boesch et al., 2011). Besides oxidative damages, DSBs are also considered as another major threat to organelle genome stability. Both plant mitochondria and chloroplast have been found to employ homology-based recombination for repairing DSBs (Li and Heyer, 2008; Maréchal and Brisson, 2010).

### Oxidative Damage in Plant Organellar Genome Is Repaired by BER Pathway

Besides performing key cellular processes, including photosynthesis and respiration, both chloroplast and mitochondria are the major sites of reactive oxygen species production. However, despite the continuous assault of oxidative damage, sequence drift in both plant mitochondrial and chloroplast genomes remains very low, indicating the presence of an efficient organellar DNA damage repair system. Recently, several studies have demonstrated that as like nuclear genome, the BER pathway also participates in the repair of oxidative DNA damages in the organellar genome (Boesch et al., 2009; Gredilla, 2010; Boesch et al., 2011; Prakash and Doublié, 2015; Peralta-Castro et al., 2020). BER in plants is a crucial genome defense pathway, comprising of sequential steps, including excision of the damaged DNA base, splitting of the sugar-phosphate backbone of DNA at the AP (apurinic/apyrimidinic) site, reorganizing the resulting DNA ends, filling of gaps through DNA replication and finally DNA strand ligation (Roldán-Arjona et al., 2019).

**FIGURE 2:**
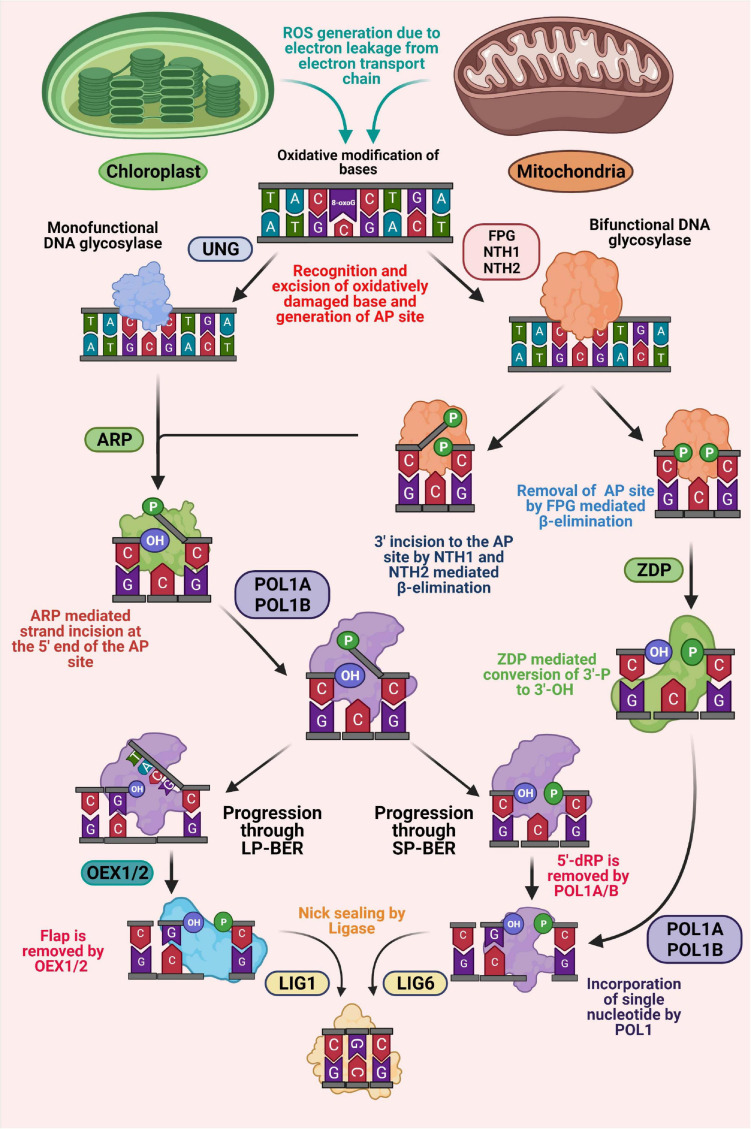
Schematic representation of base excision repair (BER) in plant organelles. BER is initiated with recognition and removal of the oxidatively modified base by the action of DNA glycosylase, generating an AP site. Monofunctional glycosylase only possesses hydrolytic activity, while bifunctional glycosylases possess an additional lyase activity. An AP endonuclease (ARP) is associated with processing the AP sites produced by monofunctional glycosylases, thus generating 3′-OH and 5′-P ends. In addition, ARP also processes 3′-OH ends produced by bifunctional glycosylases generating 3′-OH and 5′-P ends. At this point, the BER pathway diverges into short-patch (SP) and long-patch (LP) sub pathways. The 50-flap generated by the action of POL1A and POL1B during LP-BER is cleaved by FEN1 endonuclease (OEX1 and OEX2). In SP-BER, the 5′-P moiety is processed by the lyase activity of POL1s and a single nucleotide is incorporated. DNA ligase is associated with the ligation process in both LP and SP-BER pathways. In addition, in an alternate pathway, a bifunctional glycosylase, FPG, remove oxidative DNA lesion, generating 3′-P and 5′-P ends. The 3′-P end is removed by ZDP phosphatase leaving a 3′-OH end, which is subsequently resolved through BER-SP.

As in the nucleus, the BER pathway in mitochondria and chloroplast has also been found to be initiated with the action of a DNA glycosylase (Figure 2; Jacobs and Schar, 2012). Based on the substrate specificity, DNA glycosylases can be distinguished into monofunctional (only glycosylase activity) and bifunctional (glycosylase plus apurinic/apyrimidinic (AP) lyase activities) types. Monofunctional DNA glycosylases have only been found to remove the target base, thus generating an AP site. On the other hand, bifunctional glycosylases hold an additional AP lyase activity, which catalyzes an incision at 3′ of the AP site by β-elimination after base excision, thus generating 3′-α, β unsaturated aldehyde (3′-PUA), and 5′-hydroxyl (OH) termini (Figure 2; Gutman and Niyogi, 2009; Roldán-Arjona et al., 2019). Uracil-DNA glycosylases (UDG) are important monofunctional glycosylases capable of recognizing the uracil residue resulting from misincorporation of dUMP in DNA during replication or cytosine deamination and excising its N-glycosidic bond, thus generating an AP site (Roldán-Arjona et al., 2019). Previously, AtUNG (AT3G18630), a member of Family-1 UDGs has been characterized in *Arabidopsis thaliana* (Cordoba-Cañero et al., 2010). Although direct evidence of targeting AtUNG protein to the subcellular organelles, including mitochondria and chloroplast other than the nucleus have not been reported, *in vitro* import assays have revealed the transportation of UNG protein in mitochondria in *Arabidopsis thaliana*. In addition, *in vitro* targeting assays have also revealed that a transiently expressed AtUNG-GFP fusion protein in *Nicotiana benthamiana* leaves is readily colocalized with mitochondria in protoplasts generated from the agro-infiltrated tissues (Boesch et al., 2009). Moreover, some *in-organelle* experiments have further indicated the presence of UNG activity in the mitochondria in *Arabidopsis*, potato, and also in the gymnosperm *Araucaria angustifolia*, respectively (Boesch et al., 2009; Furlanetto et al., 2019).

Among various bi-functional DNA glycosylases, 8-oxoguanine glycosylase (OGG1) and the members of the formamidopyrimidine glycosylase/endonuclease VIII (FPG/NEI) family are mainly involved in repairing various oxidative damaged pyrimidines, including 8-oxoG. Previous studies have indicated that loss of FPG and OGG1 enzyme activities in *Arabidopsis thaliana* results in an increased magnitude of oxidative damage in both mitochondrial and chloroplast genome (Córdoba-Cañero et al., 2014; Chevigny et al., 2020). This strongly suggests that along with the nucleus, the BER pathway in mitochondria and chloroplast also rely on these glycosylases. Earlier investigations have revealed that oxidatively damaged pyrimidines are repaired in *E. coli* by Endonuclease III (EndoIII), also known as Nth. Two functional homologs of bacterial Nth (AtNTH1and AtNTH2) have been reported from *Arabidopsis thaliana* (Figure 2; Roldan-Arjona and Ariza, 2009; Gutman and Niyogi, 2009). Recent studies have revealed the presence of DNA glycosylase-lyase/endonuclease activity in the chloroplast protein fractions of *Arabidopsis thaliana*, thus providing meaningful evidences of involvement of DNA glycosylase-lyase/endonuclease in repairing of oxidative damaged pyrimidines through BER pathway in chloroplast (Gutman and Niyogi, 2009). *In silico* studies have identified the components of BER pathway (two endonuclease III homologs and an apurinic/apyrimidinic endonuclease) in chloroplast, which are possibly involved in this glycosylase-lyase/endonuclease activity (Gutman and Niyogi, 2009). Additional *in vitro* localization activity using transiently expressed GFP tagged proteins have further demonstrated targeting of these three proteins to the chloroplast and co-localized with the chloroplast DNA in nucleoids (Gutman and Niyogi, 2009).

Processing of blocked termini at the 5′-P and 3′-OH ends by the action of glycosylases is followed by progression of gap-filling step either by short patch (SP) or long patch (LP) BER pathway, respectively (Figure 2). Oxidative lesions processed by bifunctional glycosylases are generally repaired through the short-patch BER pathway (single nucleotide replacement). On the other hand, damages perceived by monofunctional glycosylases can be repaired alternatively either by long-patch (up to 10 nucleotide replacements) or short-patch BER (Van der Veen and Tang, 2015). Insertion of nucleotides through the SP or LP-BER pathway requires the activity of a DNA polymerase. Plant mitochondrial and chloroplast DNA polymerases have been shown to be phylogenetically linked to bacterial DNA polymerase I (Kimura et al., 2002; Shutt and Gray, 2006). In *Arabidopsis thaliana* and *Nicotiana tabacum*, two homologs of bacterial DNA polymerase I are found, which are indicated as POL1A and POL1B and shown to be targeted to both mitochondria and chloroplasts (Elo et al., 2003; Christensen et al., 2005; Mori et al., 2005; Ono et al., 2007). These plant organellar DNA polymerases have been found to possess the capability of replicating the complete organellar genome and exhibit excellent processivity without the requirement of any other accessory proteins (Takeuchi et al., 2007; Moriyama and Sato, 2014). In addition, both the polymerases have been shown to possess considerable fidelity as compared to the replicative DNA polymerases (Baruch-Torres and Brieba, 2017). Among the two plant organellar DNA polymerases, POL1A is only associated with DNA replication, whereas, POL1B is involved in DNA repair (Parent et al., 2011). Recent studies have revealed that both polymerases possess a 5′-P lyase activity, which is capable of removing 5′-P moieties formed during the short patch BER pathway (Trasvina-Arenas et al., 2018). Furthermore, it has also been found that POL1B is associated with efficient strand-displacement function on DNA containing a one-nucleotide gap, whereas POL1A shows moderate strand displacement activity, suggesting that both POL1A and POL1B can play a meaningful role in short-patch BER, while POL1B can also be involved in long-patch BER.

### Homologous Recombination Represents the Primary Mechanism for Repairing DSBs in the Organellar Genome

**FIGURE 3:**
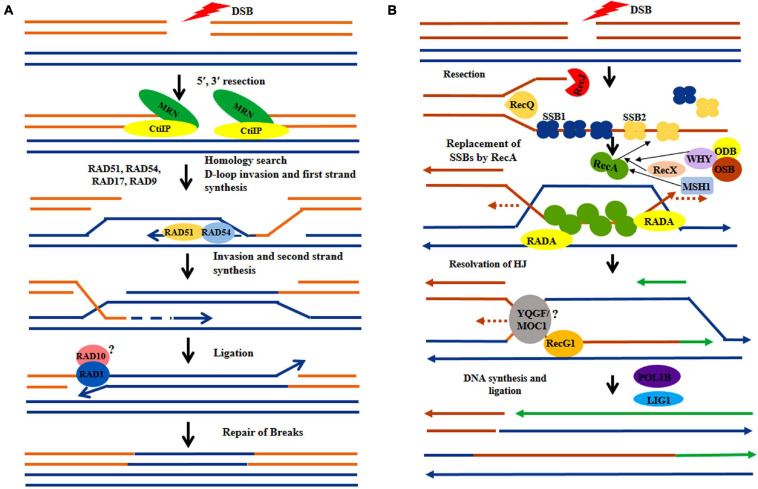
Hypothetical model comparing recombination repair in nucleus and organelles. **(A)** Following the induction of double-strand breaks (DSBs) in nuclear DNA, the DNA lesions are processed by MRN complex (MRE11, RAD50, NBS1) protein. Then the RAD52 epistatic group of genes plays a crucial role in homology search (RAD54, RAD51) and subsequent strand invasion. The DNA breaks are sealed by the joined action of polymerase and ligase, **(B)** In the sub-cellular organelles the DSB sites are processed by RecJ and RecQ nuclease proteins followed by subsequent incorporation of nuclear-encoded by Single Strand Binding proteins (SSB1, SSB2). The nuclear-encoded WHIRLY (WHY) family gene along with RecX, ODB, and OSB facilitate incorporation of RecA protein which involves in strand invasion. The uncharacterized proteins, MOC1 and YQGF may be involved in HJ resolution. The ligations of breaks are done by the joined activity of polymerase and ligase.

As in other eukaryotes, DSBs are considered as one of most crucial and deleterious forms of DNA damages in plants. When remain unrepaired, DSBs can disrupt various fundamental cellular processes, including replication and transcription. In plants, most of the DSBs in the nuclear genome are repaired by non-homologous end joining (NHEJ) or homologous recombination (HR) (Figure 3A) mediated repair pathways (Britt, 1999; Puchta, 2005; Waterworth et al., 2011; Roy, 2014). DSBs are also generated in mitochondria and chloroplast genome due to exposure to various exogenous and endogenous stress factors, such as UV-B, ionizing radiation, and the ROS continuously generated within both these organelles. Although in animal mitochondria, both HR and NHEJ pathways are involved in repairing DSBs, plant organelles possess only HR pathways, completely lacking the NHEJ repair. In addition, DSBs in higher plant chloroplast and mitochondrial DNA are also repaired by the error-prone microhomology-mediated end joining (MMEJ) repair mechanism, which represents an alternate form of NHEJ repair (Kwon et al., 2010; Seol et al., 2017).

Homologous recombination is important for the repair of DSBs in the organellar genome and has generally been found to occur by the invasion of a homologous DNA strand followed by strand exchange (Figure 3B; Li and Heyer, 2008). Homologous recombination plays a crucial role in mitochondrial DSB repair and loss-of-function of any of the HR-associated protein results in significant rearrangements in the mitochondrial genome (Davila et al., 2011). Under genotoxic stress conditions, plant mt-DNA exhibits higher recombination frequency (Kuehn and Gualberto, 2012). Presence of large inverted repeats in the chloroplast genome provides increasing chances of availability of homologous sequences for homologous recombination, which involves repair of DNA lesions via gene conversion (Wolfe et al., 1987; Muse, 2000; Gualberto and Newton, 2017). Chloroplast employs all types of HR repair mechanisms, including double-strand break repair (DSBR), single-strand annealing (SSA), and synthesis-dependent strand annealing (SDSA) for DSB repair (Kohl and Bock, 2009). It has been suggested that because of their high homologous recombination frequencies, both mitochondrial and chloroplast genomes show a comparatively slower rate of evolution with a reduced substitution rate. In angiosperms, the rate of synonymous substitution in mitochondria, chloroplast, and nuclear genes appears as 1:3:10, exhibiting their high efficiency in genome repair (Drouin et al., 2008).

Two families of plant-specific single-strand binding proteins, including organellar single-strand binding proteins (OSBs) and plant-specific WHIRLY family proteins, are involved at the initial phase of homologous recombination in both mitochondria and chloroplast (Maréchal and Brisson, 2010). These proteins are encoded by the nuclear genome and have been targeted to either mitochondrion (OSB1, OSB4, and WHY2) or chloroplast (OSB2, WHY1, and WHY3) or both (OSB3) through anterograde signaling (Maréchal and Brisson, 2010; Garcia-Medel et al., 2019). All OSBs have been found to possess an N-terminal nuclear targeting peptide for their convenient targeting to the respective organelles. Although, not highly well conserved (30–50% similarity) in terms of amino acid residues, a domain having significant structural similarities with the oligonucleotide/oligosaccharide binding fold (OB-fold), a distinctive feature of many ssDNA-binding proteins, is has been found in the internal region of OSBs. Among various WHIRLY family proteins, chloroplast targeted WHY1 and WHY3 have been found to protect chloroplast genome from rearrangements (Mare’chal et al., 2009). On the other hand, WHY2 is targeted to mitochondria and is involved in strand resection following oligomerization (Maréchal and Brisson, 2010).

The bacterial recombinase RecA and its eukaryotic homologs of the Rad51 family are essential proteins that have been shown to play a central role in homologous recombination and thus are crucial for genome stability maintenance (Cox, 2007; Odahara et al., 2015). They are key factors involved in the accurate pairing of homologous DNA sequences, promotion of strand invasion, and branch migration during the recombination process. In bacterial recombination-based repair, the exonuclease activity of RecBCD helps in resection of the 5′ phosphate after induction of DSBs. Then RecA is involved in the formation of the presynaptic complex (Handa et al., 2009). Nucleation of RecA occurs in a RecFOR in protein-dependent manner and the dynamic nature of RecA initiates the homologous sequence finding process (Sandler and Clark, 1994). Plant nuclear genome characteristically encodes three bacterial−type RecA homologs, including RecA1, RecA2, and RecA3, which are localized in chloroplasts and/or mitochondria, in contrast to their nuclear counterpart Rad51. Among the three RecAs, RecA1 and RecA3 are specifically targeted to chloroplast and mitochondria, respectively. On the other hand, RecA2 is targeted to both, either chloroplast or mitochondria (Shedge et al., 2007; Odahara et al., 2009; Chevigny et al., 2020). Knockout of mitochondria-targeted RecA3 has resulted in large-scale rearrangements in the mitochondrial genome (Odahara et al., 2015). Besides higher plants, RecA1 has also been reported from *Physcomitrella patens* (moss) and disruption of PpRecA1 has been shown to cause considerable sequence rearrangements involving short repeats (Small, 1987; Odahara et al., 2009). The molecular function of RecA2 in homologous recombination in the organellar genome is further regulated by RecX protein in plants. The *recA3odb1* and *recA3msh1*double mutants are shown to exhibit developmental abnormalities (Chevigny et al., 2020). Therefore, it is suggested that organelle-targeted RecA homologs are involved in preventing recombination between short repeated sequences and thus promoting DNA damage repair.

The second branch point in the bacterial genome occurs in a RuvABC (helicase) mediated way. RuvABC has been shown to play a key role in branch migration during SDSA or SSA repair in bacterial cells. Although, no homolog of bacterial RuvABC is found in plant mitochondria, RadA and RecG proteins have been found to play a similar role in plant mitochondria (Whitby et al., 1994; Marie et al., 2017; Chevigny et al., 2019). RecG is involved in the migration of the holiday junctions to the very end of the DNA molecule and is also involved in D-loop formation. The activity of RecG further converts the D-loop to a holiday junction and subsequently, the endonucleases cleave the heteroduplex to recover the two sister chromosomes. The homolog of RecG protein is identified in both mitochondria and chloroplast (Wallet et al., 2015; Odahara et al., 2017). A knockout mutation of RecG results in an ectopic recombination event in mitochondria with aberrant organellar structure (Odahara et al., 2017). RadA, also involved in branch migration, is a paralog of RecA and has already been characterized in *Arabidopsis thaliana* (Chevigny et al., 2020). Knockout of this gene in chloroplast and mitochondria results in the arrest of plant growth and development along with permanent impairment of mitochondrial genome stability (Chevigny et al., 2020). Although no functional organellar protein with resolvase activity has been reported, a nuclear-encoded protein is specifically recruited to chloroplast, which appears to play a similar function to bacterial RuvC resolvase (Wardrope et al., 2009; Kobayashi et al., 2017). In addition, monokaryotic chloroplast 1 or MOC1 has been found to resolve the holiday junction in the chloroplast genome, and absence of this protein may result in aberrant chloroplast segregation and complete loss of cp-DNA (Kobayashi et al., 2017). Together these pieces of evidences have indicated the similarity of bacterial and organellar homologous recombination processes, which also further supports the notion of the endosymbiotic origin of the organelles.

A MutS homolog, MSH1 was identified in both chloroplast and mitochondria. Beside their precise role in recognition of DNA damage associated with mismatches, plant MSH1 also contain a C-terminal endonuclease domain, GIY-YIG, which introduces DSBs and recruit homologous DNA damage repair machinery for accurate repair. Thus, the function of MSH1 is more than a mismatch repair protein in both chloroplast and mitochondria (Abdelnoor et al., 2003). In *Physcomitrella patens* (moss), duplicated genes of MSH1, MSH1A, and MSH1B are present. Among these two, MSH1B genetically interacts with RecA2 and RecG to maintain organellar genome stability (Odahara et al., 2017). Furthermore, the *msh1breca2* and *msh1brecg* double mutants have been shown to increase recombination between short dispersed repeats (SDRs) in chloroplasts and mitochondria synergistically (Odahara et al., 2017).

The organellar DNA not always requires long homologous repeats to repair DSBs. It has been reported that chloroplast DNA in *Arabidopsis thaliana* may also repair DSBs via microhomology-mediated end joining (MMEJ), distinct from homologous recombination (HR) (Kwon et al., 2010). MMEJ is a sub-type of alternative non-homologous end-joining (A-NHEJ) mechanism that requires very short regions (2-14 bp) of homology. In this process, DSBs have sealed microhomology (MH)-mediated base-pairing of DNA single strands, followed by the steps including nucleolytic trimming of DNA flaps, DNA gap filling, and DNA ligation (Seol et al., 2017). Although this process is error-prone, but possibly have further contributed to land plant genome evolution (Heacock et al., 2004; Kwon et al., 2010).

### Coordination of Nuclear and Organellar Genome via Inter-Compartmental Crosstalk Is Crucial for the Maintenance of Organelle Genome Stability in Plants

Plants require the coordinated action of all three genomic compartments for their harmonious growth and development. The endosymbiotic origin of mitochondria and chloroplast and the subsequent transfer of a significant amount of genetic material to the nuclear compartment through horizontal gene transfer leaving few active genes to be encoded by the organellar genome (Pfannschmidt and Yang, 2012; Nakashima et al., 2014). Proteins associated with various cellular functions in plants are found to be encoded by the genome of different compartments. So, there must be a coordinated mechanism existing between various organelles within the plant cell that is involved in regulating the inter-organellar crosstalk during organellar biogenesis or stress responses (Busi et al., 2011; Ng et al., 2014). The inter-organellar crosstalk is of two types, including anterograde (nucleus to organelle) and retrograde (organelle to the nucleus) signaling (Busi et al., 2011; Bobik and Burch-Smith, 2015). Anterograde signaling initiates from the nucleus, appears to sense both the endogenous and exogenous environmental cues that may lead to modulate the expression of various mitochondrial and chloroplast genes. On the other hand, the retrograde mechanism is found to rely on the physiological state of organelles, which regulates the expression of nuclear genes (Ng et al., 2014; Chan et al., 2016; Börner, 2017). The balance of the protein pool in the nucleus and organelles can be modulated by two primary mechanisms. One of these mechanisms is dual targeting from the cytoplasm to either nucleus or organelles. Another one is targeting organelles and then subsequent relocation to the nucleus (Krause and Krupinska, 2009). Recently, various pathways have been identified that are probably involved in retrograde signaling including genome uncoupled (GUN) loci mediated chloroplast to nucleus signal transduction pathway, SAL1-PAP pathway, methylerythritol mediated pathway, and carotenoid mediated pathway, respectively (Strand et al., 2003; Cordoba et al., 2009; Estavillo et al., 2011; Giuliano, 2014). In yeast and animal, the inter-genomic signals between mitochondria and the nucleus not only regulate the mitochondrial biogenesis but also modulate gene expression throughout different developmental stages (Poyton and McEwen, 1996; Hess and Bo”rner, 1999). In mitochondria, the oxidized guanines (8-oxoG) are repaired by a specific bi-functional glycosylase, OGG1 (8-oxoguanine glycosylase) through base excision repair pathway (Córdoba-Cañero et al., 2014). It was found that in humans, the *OGG1* gene is differentially spliced at exon 7 and exon 8 and directed to both nucleus and mitochondria, respectively (Seeberg et al., 2000). In plants also, genomic compartments including, mitochondria and chloroplast are shown to be dependent on the nuclear genome and most of the proteins have been imported post-translationally during the growth and development (Zhang et al., 2011; Krupinska et al., 2020). The proteins targeted to mitochondria or chloroplast generally possess an N-terminal mitochondrial or chloroplast targeting signal, which enables them to be translocated through the outer and inner membranes (TOM, TIM; TOC, TIC) of chloroplast and mitochondria (Richly and Leister, 2004; Melonek et al., 2012; Saki and Prakash, 2017).

In plants, organelles act as the intracellular sensors of stress cues and convey the signal to the nucleus, followed by activation of an array of molecular signals. Efficient regulation of energy-producing reactions in organelles and inter compartmental crosstalk following various abiotic stresses is essential for maintaining cellular homeostasis and also survival of plants (Jazwinski, 2013; Cagin and Enriquez, 2015). In animal cells, after induction of DNA damage, different signaling networks, including Ca^2+^, NAD^+^/NADH, AMPK, redox signaling, and mitochondrial unfolded protein response (UPR) mediated pathways have been found to be activated (Janssen-Heininger et al., 2008; Rizzuto et al., 2012; Stein and Imai, 2012; Tripathi et al., 2013; Jovaisaite et al., 2014). Introducing mutation in the nuclear gene *DAG* in *Antirrhinum majus* and *DCL1* in tomato has been shown to hamper chloroplast development, resulting in small chloroplasts with impaired internal membrane system (Keddie et al., 1996). The interdependence of chloroplast and mitochondria has already been proven from the loss-of-function mutants of non-chromosomal striped (NCS) in maize containing deletions in *COXII*, *NAD4*, and *NAD7* genes at coding regions (Li et al., 2018). Furthermore, albostrian barley with impaired photosynthetic activity and chloroplast development showed elevated expression of several mitochondrial genes encoding cytochrome c oxidase and ATPase subunits, including *COXII*, *COXIII*, *ATPA*, *ATP6* and *ATP9*, respectively (Hedtke et al., 1999). Defects in plant mitochondrial glycine decarboxylase have resulted in developmental deformities in chloroplast, including excessive excitation and reduction (Sabar et al., 2000; Heineke et al., 2001). Together these investigations have suggested the interdependence of organelles and the existence of a strong inter compartmental signaling network within plant cell.

**FIGURE 4:**
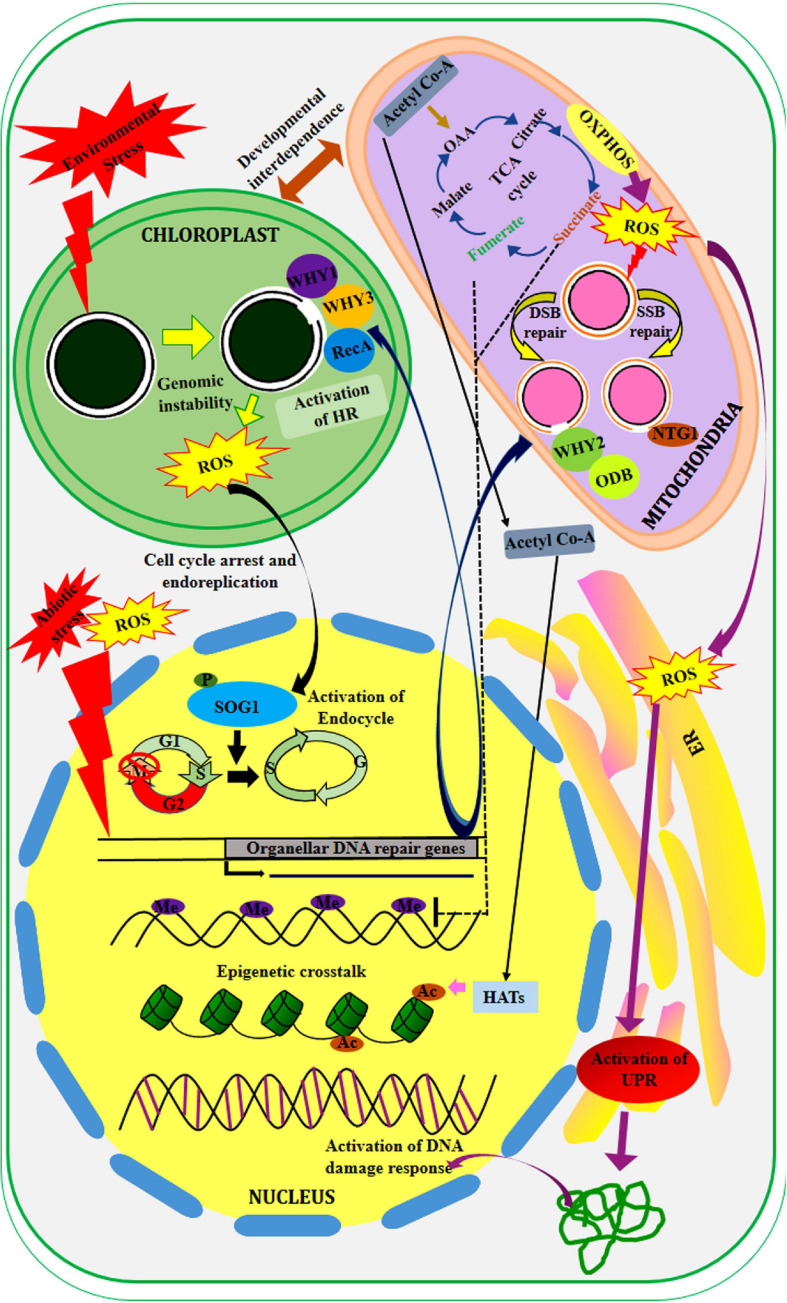
Inter-compartmental crosstalk in the plant cell. The organelles are developmentally interdependent. The mutation of chloroplast genes affects the expression of several important genes (not shown here). In response to different environmental stress, the chloroplast genome become unstable which leads to the production of ROS in this compartment. This signal is received by the transcription factor, SOG1 that promotes endoreduplication and cell cycle arrest in the nucleus. While the nuclear-encoded proteins related to organellar DNA repair of both single and double-strand break repair are transported to the chloroplast (WHY3, WHY1, and RecA) and mitochondria (WHY2, ODB, NTG1) via anterograde signaling. The products of the TCA cycle (Succinate and fumarate) epigenetically modulate the gene expression via activation or repression. Moreover, the metabolic by-product (ROS) generated in mitochondria activates unfolded protein response (UPR) in ER which subsequently promotes DNA damage response in the nucleus.

Plethora of studies has revealed that retrograde signaling is activated in plants under different biotic and abiotic stress conditions (Figure 4; Huang et al., 2003). In *Arabidopsis thaliana*, loss-of-function of IM genes in nuclear genome encoding a terminal oxidase in chloroplast has resulted in overproduction of ROS and subsequent oxidation of genomic elements (Foudree et al., 2012). Another study has shown that *CHM* (chloroplast mutator) gene in *Arabidopsis* encodes a mitochondrion targeted protein, which is associated with maintaining mitochondrial genome stability and integrity (Abdelnoor et al., 2003). In yeast, NTG1, a bi-functional DNA glycosylase with lyase activity, possess both nuclear localization signal and mitochondrial matrix targeting sequence and is found to be located both in mitochondria and nucleus (Swartzlander et al., 2010). During excessive oxidative stress, NTG1 has been shown to modulate the stress response and activate base excision repair pathway in mitochondria. Oxidatively damaged pyrimidines are repaired in *E. coli* by Endonuclease III (EndoIII), also known as Nth. Two functional homologs of bacterial Nth (AtNTH1and AtNTH2, respectively) have been characterized from *Arabidopsis thaliana* (Roldan-Arjona and Ariza, 2009). Recent studies have revealed the presence of DNA glycosylase-lyase/endonuclease activity in the chloroplast protein fractions of *Arabidopsis thaliana* (Gutman and Niyogi, 2009). In yeast, fumarase, a mitochondrial genome encoded protein that converts fumarate to malate, is also involved in the activation of DNA damage repair in the nucleus by inhibiting histone H3 demethylation (Jiang et al., 2015). Proteins encoded by the *WHIRLY* (*WHY*) family of genes in the nuclear genome act as organellar single-strand binding proteins and can be targeted to either chloroplast or mitochondria or both (Zaegel et al., 2006). These proteins play a crucial role in the initial stages of recombination-mediated repair in organellar genomes. In *Arabidopsis thaliana*, two *WHIRLY* family genes; *AtWHY1* and *AtWHY3* are specifically targeted to chloroplast where they suppress chloroplast genome rearrangements (Mare’chal et al., 2009). On the other hand, *AtWHY2*, another member of this family is targeted to mitochondria. In *Arabidopsis thaliana*, among the six SWIB (SWI/SNF complex B) domain-containing proteins, four proteins are found to be targeted to chloroplast and associated with the chloroplast nucleoids. Among these chloroplast-targeted SWIB members, SWIB-6 and SWIB-4 have been shown to exhibit a second localization in mitochondria and nucleus, respectively (Melonek et al., 2012). The recombinant SWIB-4 has been shown to influence the compaction and condensation of nucleoids. In *Arabidopsis*, DNA ligase I (AtLIG1) is found to be located in both nucleus and mitochondria along with type I and type II isomerases (Sunderland et al., 2006; Moriyama and Sato, 2014). The AtLIG1 activity is modulated in different cell types and metabolic states. Besides the role in ligation, this mitochondrial targeting protein is also involved in excision repair (Sunderland et al., 2006). Initial studies have reported a complete absence of photoreactivation in plant organelles (Chen et al., 1996; Hada et al., 2000). However, in rice and *Arabidopsis*, a nuclear-encoded photolyase was detected in both mitochondria, and chloroplast, respectively (Kleine et al., 2003; Takahashi et al., 2011). A single Mismatch repair (MMR) protein, MSH1, is nuclear-encoded and targeted to both chloroplast and mitochondria to repair the mismatches and also facilitates the recruitment of HR repair proteins in organelles (Chevigny et al., 2020).

Besides the DNA repair proteins, several transcription factors have been shown to play crucial role in anterograde signaling, where they participate in regulating the expression of different stress-responsive mitochondrial and chloroplast genes (Figure 4). In plants, a NAC domain transcription factor SOG1 (SUPPRESSOR OF GAMMA RESPONSE 1), the functional homolog of mammalian p53, has been found to regulate various aspects of DNA damage response in the nucleus, including DNA damage repair, cell cycle checkpoint control, and endoreduplication for ensuring genome integrity and stability (Adachi et al., 2011; Yoshiyama et al., 2017; Mahapatra and Roy, 2020). SOG1, the central regulator of DNA damage responses in plants, is also found to mediate endoreplication and cell-cycle progression through retrograde signaling from chloroplast to nucleus in response to chloroplast genome instability. *Arabidopsis thaliana* triple mutant *recA1why1why3* with considerable chloroplast genome instability have shown elevated expression of *RAD51* and *KU70*, the DNA repair marker genes associated with nuclear DSB repair (Horvath et al., 2017). In the same triple mutant, upregulation of various genes associated with cell cycle inhibition including *SMR5* and *SMR7* has also been detected, indicating arrest of cell cycle and induction of endoreduplication in response to chloroplast genome instability. Possibly, this response is regulated by SOG1, as all these genes are the direct target of SOG1 (Ogita et al., 2018; Mahapatra and Roy, 2020; Duan et al., 2020). This probably showcases a novel example of a retrograde signaling pathway, where nuclear gene expression is modulated by organellar genome instability and dysfunction (Chi et al., 2013; Hudik et al., 2014).

## Epigenetic Modifications Are Involved in Maintaining the Stability of the Organellar Genome

Epigenetic changes affect an array of cellular processes in both prokaryotes and eukaryotes and these changes are not associated with permanent alterations in DNA sequence, while modify gene expression. Such changes are still heritable to the next generation (Kinoshita and Seki, 2014). Epigenetic modifications are generally caused by post-translational modifications (PTMs) that exist in the nuclear genome, including methylation, acetylation, phosphorylation, ubiquitination, PARylation, and sumoylation of protein. In addition, methylation of mainly cytosine residues in the promoter and intergenic regions also represents another important epigenetic modification in plants. These modifications are collectively involved in mediating various biotic and abiotic stress responses in plants (Bannister and Kouzarides, 2011; Kim, 2019). Besides these post-translational modifications, recent studies have revealed that different non-coding RNAs are also involved in epigenetic regulation (Agarwal and Miller, 2016). In plants, two subcellular organelles, mitochondria and chloroplast with self-contained genetic material have been found to coexist with the nucleus. However, very little information is available in the context of epigenetic modification and regulation of organellar genomes in plants.

### Epigenetic Regulation by Post-translational Modifications

Among the various forms of post-translational modifications, cytosine DNA methylation has been identified as one of important heritable epigenetic marks found in many eukaryotes. Although DNA methylation is found to perform a conserved role in gene silencing, the levels and patterns of DNA methylation appear to differ significantly among different organisms. Methylation of DNA bases, as well as N and C-terminal tails of histone proteins modulate the nuclear gene expression. Cytosine residues are the major targets of methyltransferases (Methylation enzyme), which convert cytosine to 5-methyl cytosine (Richards, 2006). Methylation has also been found to play crucial role in organellar genome maintenance in plants (Gauly and Kössel, 1989). DNA methylation in the organellar genome has been a topic of research for past couple of decades. Although organellar DNA generally remains unmethylated, some of the cytosine residues in chloroplast DNA in the unicellular green alga *Chlamydomonas reinhardtii* have been found to be methylated (Dyer, 1982; Sager et al., 1984). During reproduction in *Chlamydomonas reinhardtii*, the chloroplast DNA gets degraded due to activation of zygote maturation process (Umen and Goodenough, 2001). However, methylation has been found to prevent the degradation of chloroplast DNA during mating (Sager et al., 1984; Umen and Goodenough, 2001). Subsequently, cytosine methylation in plastome was also reported in many higher plants, including *Acer*, tomato, and maize (Ngernprasirtsiri et al., 1988a,b; Gauly and Kössel, 1989). Leaf chloroplast in rice becomes more methylated with aging (Muniandy et al., 2019). In addition, the differential methylation pattern of CpG and CHG islands could be detected in leaf chloroplast and grain amyloplast of rice with identical DNA sequences (Muniandy et al., 2019). Besides chloroplast, a more recent study has indicated cytosine methylation in rice mitochondrial genome (Muniandy et al., 2020). In rice, leaf mitochondrial DNA showed higher proportions of methylation as compared to the grain mitochondrial DNA (Muniandy et al., 2020).

Cytosine methylation in plants can occur at different sites, like CG, CHG, and CHH, where H indicates nucleotide other than G (Law and Jacobsen, 2010). Earlier studies have indicated a tissue-specific organellar methylation pattern of cytosine residues, while in some cases methylation was completely absent (Yan et al., 2010). Furthermore, the same study has also revealed the presence of about 22 methylation sites in the coding region of the organellar DNA and about 146 positions dispersed in intergenic parts, indicating the variation in the position of methylation (Yan et al., 2010). Though nuclear methylases have been well characterized in plants, the information on methylation mechanism via specific methylases in organellar genome remains mostly inadequate. Previous studies have employed cyanobacterial methylase for the characterization of epigenetic regulation in plant chloroplast (Ahlert et al., 2009). In the nucleus, DNA methylation of cytosines has been found to occur specifically in CpG islands, which are dinucleotide repeat elements of typically 500-1500 bp length. In mitochondria, the story is somehow different as the CpG islands are absent in the mitochondrial genome due to their relatively smaller size (Muniandy et al., 2020).

Organellar genomes also affect the intricacy of the plant nuclear genome. Chloroplast and mitochondrion are the salient plant organelles with their own genetic material. During the course of evolution, most of the genetic information of organelles have been transferred to the nucleus and subsequently integrated with the nuclear genome, leading to the formation of NUPTs (Nuclear integrant of plastid DNA) and NUMTs (Nuclear integrant of mitochondrial DNA) (Yoshida et al., 2019; Zhang et al., 2020). In plants, NUPTs are shown to be involved in centromere formation and sex chromosome evolution (Li et al., 2019). The regulation and expression of integrated organellar DNA fragments in the nucleus are controlled via methylation. Considerable decrease in methylation level of NUPTs has been detected during evolution after being integrated into nuclear genomes (Paszkowski and Whitham, 2001; Bender, 2004). Thus, DNA methylation on nuclear organellar DNA fragments may also contribute toward the maintenance of genome stability and evolutionary dynamics of symbiotic organellar as well as their host’s genomes. Although, there has been no direct evidence of plant organellar genome methylation, methylome data analysis using various epigenetic mutants have revealed the establishment and maintenance of methylation of NUPTs (Nuclear integrant of plastid DNA) in *Arabidopsis thaliana* by several methyltransferases including DDM1, CMT3, CMT2, and SUVH4/KYP proteins, respectively (Yoshida et al., 2019; Zhang et al., 2020). In the absence of histone proteins, mitochondrial gene expression is regulated by the epigenetic changes of nucleoid-associated proteins. In animal system, multiple lysine acetylation and phosphorylation sites have been detected in the organellar genome (Zhao et al., 2010). Mitochondrial transcription factor A (TFAM) has been shown to be post-transcriptionally modified via glycosylation, acetylation as well as phosphorylation (Lu et al., 2013; King et al., 2018). However, corresponding information in plant mitochondria genome remains much limited and further investigation is required. In humans, poly ADP ribosylation is considered as one of the key post-translational modifications regulated by poly ADP ribose polymerases (PARP). Three canonical PARP proteins are encoded by *Arabidopsis* nuclear genome (Gu et al., 2019). Among the three PARP proteins, PARP1 has been found to be conserved throughout the plant and animal kingdom, while PARP2 proteins are broadly conserved across diverse plant groups. In contrast, PARP3 is mainly expressed in seed tissues in plants, but can also be detected in the seedlings and roots in adult plants (Feng et al., 2016; Rissel and Peiter, 2019). In *Arabidopsis*, AtPARP2 plays a predominant role in poly-ADP ribosylation than AtPARP1. AtPARP2 is localized in the nucleus, as evidenced from the expression of *AtPARP2-GFP* constructs in *Nicotiana benthamiana* and *Arabidopsis* (Song et al., 2015; Pham et al., 2015; Chen et al., 2018). Recent studies have demonstrated that nuclear import of AtPARP2 is mediated via importin-α (Chen et al., 2018). In addition to its nuclear localization, AtPARP2 has been suggested to be partially localized in chloroplasts (Pham et al., 2015). As with AtPARP2, the AtPARP1-GFP fusion protein was detected in nucleus in *Nicotiana benthamiana* and in *Arabidopsis* cell suspension culture (Song et al., 2015; Pham et al., 2015). Furthermore, PARP1-GFP localization was detected in chloroplasts and mitochondria when expressed in *Arabidopsis* protoplasts (Pham et al., 2015). In plants, PARP2 regulates response to various genotoxic agents such as bleomycin, mitomycin-c, or γ-radiation, respectively (Song et al., 2015). Nuclear encoded PARP2 also regulates plant immunity via interacting with fork head associated (FHA) domain protein, DAWDLE (DDL) (Feng et al., 2016). Recent studies have revealed that PARP1 is located both nucleus and mitochondria (Pankotai et al., 2009). PARP1 binds to the promoter of several mitochondrial-targeted nuclear protein-encoding genes, which are specifically involved in mitochondrial DNA damage repair. PARP1 also modulates the expression of many DNA damage repair genes, including *APE1*, *UNG1*, *MYH1*, and various transcription factors including TFB1M and TFB2M, respectively (Lapucci et al., 2011).

### Epigenetic Regulation of Organellar Genome by Non-coding RNAs and MicroRNAs

Besides the precise role of cytosine methylation in the regulation of epigenetic responses in the organellar genome, several non-coding RNAs are also found to be involved in the regulation of expression of organellar proteins encoded by nuclear genes (Lung et al., 2006). Intra-organellar non-coding RNAs also alter the expression of both mitochondrial and chloroplast genes (Zhang et al., 2016; Matsui and Corey, 2017). These non-coding RNAs are either synthesized *de novo* via the transcription of mitochondrial or chloroplast genome or may be generated from the degradation of the organelle transcriptome, as it was evidenced from Pentatricopeptide Repeat Proteins (PPR) (Hotto et al., 2012). Various reports have claimed the location of intermediate size non-coding RNAs in the protein-coding genes, including *ATP*, *RPL*, *NAD*, and *COX3*, respectively in *Arabidopsis thaliana* (Wang Y. et al., 2014). In barley grains and leaves, exonic regions of mitochondrial DNA produce different circular RNAs and their expression varies in response to different micronutrients and they are also involved in an array of metabolic pathways (Darbani et al., 2016). Previous studies have revealed abiotic stress mediated up-regulation of various microRNAs (miRNAs) in chloroplasts and among which, mi-408 was found to be highly induced under cold stress (Liu et al., 2008). However, the information regarding involvement of various non-coding RNAs in regulating DNA damage response in plant organellar genome remains largely unknown. Therefore, further studies are required for the characterization of role of different non-coding RNAs in organellar genome maintenance.

Besides non-coding RNAs, various miRNAs are also found to be associated with several biological processes, including animal cell development and differentiation. It has been observed that miR125a is involved in the sex-determination and development of mammalian gonads (Reza et al., 2019). Many human diseases are associated with the aberrant expression of miRNAs (Paul et al., 2018). Moreover, muscle-specific miR-1 is capable of enhancing the expression of mt-DNA encoded proteins during muscle differentiation, which also requires argonaut 2 (AGO2) (Zhang et al., 2014).

## Targeting Organellar Genome for Crop Improvement

The detrimental effects of global climate change along with the augmented increase in human population have put a substantial impact on agriculture and thus, emphasis has now been given toward ensuring food security for a sustainable future (Foley et al., 2011; Tilman et al., 2011; Verma et al., 2020). Conventional plant breeding for generating plants with high productivity is often found to be a comparatively labor-intensive and time-consuming procedure as compared to plant genetic engineering approaches, which is believed to enhance crop productivity more efficiently (Christou, 2013).

Genetic diversity is one of the very essential components for crop improvement to generate novel combinations of genes to achieve desired phenotypes (Glaszmann et al., 2010). Although DNA damage has been considered for its mutagenic effect, the persistence of damaged bases in DNA also harms plant growth and development. Due to their immobile nature and obligatory dependence on sunlight for photosynthesis, as like the other plants, crop plants’ genome also remains continuously exposed to various genotoxic factors, including solar UV and ionizing radiation, soil salinity, heavy metal contamination and also the by-products of endogenous metabolic processes, such as ROS, which may frequently result in the accumulation of spontaneous mutations (Verma et al., 2020). Although these mutations have been found to enrich the genetic diversity in the already existing genetic pool, the process is too slow to keep pace with the ever-increasing demand. At present, various crop improvement tools are used, which can be broadly categorized into major three types, such as the chemical and physical mutagenesis, transgenics, and genome editing approaches (Manova and Gruszka, 2015; Verma et al., 2020). Intriguingly, DNA damage response or DDR has taken the central stage in almost all crop improvement techniques. Techniques of mutagenesis appeared to be one of the effective tools for developing various germplasm collections in both model and crop plants and have facilitated the discovery of desired loci and alleles (Manova and Gruszka, 2015). Significant advancement has been made for the past couple of years to decipher the underlying mechanisms of organellar genome stability maintenance in plants. Further identification and functional characterization of different genes involved in these processes therefore may provide important insight into the molecular mechanisms of DNA repair. Induction of mutations in the crucial genes involved in DNA damage response and repair in organellar genomes have been found to alter the efficacy of these important processes, resulting in more effective mutagenesis (Verma et al., 2020).

Nuclear genome transformation has been employed extensively to increase productivity of some economically important crop plants (Li et al., 2021). However, one of the major constraints of nuclear transformation is the uncertain expression of the gene of interest along with the random location of insert DNA integration, resulting in gene silencing (Meyers et al., 2010). In this context, the presence of highly efficient homology-dependent DSB repair in the organellar genome has opened up new possibilities in the area of genome transformation and overall crop improvement (Li et al., 2021). Along with the highly efficient homologous recombination process for precise transgene insertion, some other attractive characteristics, including high intensity of transgene expression, multiple transgenes stacking through operon transfer to the organellar genome, absence of epigenetic regulation mediated gene silencing, and expression of native bacterial proteins, respectively have provided a better option for settlement of transgene within the organellar genome (Bansal and Saha, 2012; Li et al., 2021). Chloroplast transformation has been successfully performed in the model plant *Arabidopsis thaliana* (Ruf et al., 2019). However, successful transformation of mitochondria in higher plant systems has not been reported so far. In crop plants, chloroplast transformation has been limited due to technical difficulties. However, it has been suggested that the development of species-specific transgene delivery vectors with homologous recombination sequences along with regulatory elements for methodical transgene integration would effectively facilitate chloroplast transformation (Bansal and Saha, 2012).

### Targeting DNA Damage Response and Repair System in Subcellular Organelle for Crop Improvement

Plants have evolved with vast array of DNA damage response and repair systems for efficient detection and repair of the damages in the genome for maintaining genome stability and to cope up with various genotoxic threats. However, information regarding mechanisms of DNA damage repair in plant organellar genome and their implications is rather limited. Various genes involved in DDR are known to be responsive to different abiotic and biotic stress factors and targeting these genes by altering their expression or protein structure may help in generating improved stress tolerance in crop plants (Baillo et al., 2019). Many DNA glycosylases involved in repairing oxidative damages, including the repair of one of the highly mutagenic lesions, 8-oxo guanine (8-oxoG) via base excision repair (BER) pathway and have been shown to play an essential role in developing oxidative stress tolerance. In *Arabidopsis*, overexpression of *AtOGG1* (codes for AtOGG1 DNA glycosylase) improves seed longevity (Chen et al., 2012) while, overexpression of *AtMBD4* (codes for MBD4L DNA glycosylases) has been shown to enhance tolerance to oxidative stress in *Arabidopsis* (Nota et al., 2015). Studies carried out with the nuclear genome have provided important clues regarding the altered expression of DNA glycosylases associated with the BER pathway in mitochondria and chloroplast for developing enhanced tolerance to biotic and abiotic stress factors in plants. Therefore, it would be highly interesting to understand the functional aspects of DNA glycosylases associated with organellar base excision repair to get further insight into the potential scope of utilization of BER pathway components in crop improvement.

Although, the homology-dependent DNA repair has been primarily demonstrated in nuclear and prokaryote genome, accumulating evidences have revealed the presence of fairly well-developed homologous recombination pathway in both mitochondria and chloroplast in many plant species (extensively reviewed by Barr et al., 2005; Kmiec et al., 2006). Frequent and easily noticeable flip-flop recombination events between large inverted repeats could be detected in chloroplasts of many angiosperms, resulting in equimolar plastome isoforms with different orientations of the two single-copy regions (Palmer, 1983). Homologous recombination can be employed as a fundamental and potential driving force for the generation of new allelic combinations as well as enhancement of genetic diversity. Thus, understanding the in-depth mechanism of homologous recombination and its possible interlinking with other pathways of the organellar DNA damage response may provide an effective tool for modulating HR frequencies for achieving the goal of crop improvement. In addition, it may also provide one possible mechanism to ease the homologous recombination between divergent sequences.

### Chloroplast Genome Editing for Genetic Analysis

The present nuclease based genome editing approach relies on the induction of DSBs at the targeted DNA sequences of targeted genome alterations in the context of crop improvement (Puchta and Fauser, 2015; Schmidt et al., 2019). The resulting DSBs can be repaired by both the non-homologous end joining (NHEJ) and homologous recombination (HR) mediated repair pathways. However, the skewness of the former pathway normally results in imprecise repair as well as random mutations (Ray and Raghavan, 2020). On the other hand, the homology-directed repair has been considered as one of the most error-free pathway as it involves a precise sequence alteration based on a homologous DNA sequence as template (Chen et al., 2019). In plants, in both mitochondria and chloroplast, DSBs are mainly repaired via homologous recombination and microhomology-mediated end joining pathway, while the NHEJ repair pathway appears to be either completely absent in plant organellar genome or may be present in few cases (Kwon et al., 2010). In the chloroplast of the green algae *Chlamydomonas*, all three forms of HR pathways, including DSBR, SDSA, and SSA have been reported (Odom et al., 2008; Kwon et al., 2010). CRISPR-Cas9-mediated genome alteration approach was applied in *Chlamydomonas* by introducing two plasmids. One of the plasmid was used for carrying Cas9 and a guide RNA (gRNA) expression cassette, while the other plasmid carried the precise donor DNA fragment for incorporation between the two specific DSB sites created by the targeted action of CRISPR-Cas9 (Yoo et al., 2020). Interestingly, at one of the Cas9 incision sites in the *Chlamydomonas* chloroplast, no insertions or deletion could be detected, indicating the absence of error-prone NHEJ repair pathway in the *Chlamydomonas* chloroplast genome. However, the possibility of activation of the CRISPR-Cas9 system in the chloroplast of *Chlamydomonas* cannot be discarded. For now, one of the major constraints for employing the CRISPR-Cas9 genome editing system to manipulate organellar genomes in higher plants is the presence of double-membrane boundary of the organelles, which efficiently restricts the import of various biomolecules, including the nucleic acids (Gammage et al., 2018). One of the possible approaches for genome editing in chloroplast may involve direct expression of both Cas9 and gRNA in the chloroplast genome by using the privilege of chloroplast transformation technology. However, one of the major limitations in this approach is the high copy number of the chloroplast genome (Shaver et al., 2006). Possible risk of gene conversion between wild-type and disrupted genome may occur in such condition if the chloroplast genome with insertion and/or deletions, generated by CRISPR-Cas9, fails to achieve a homoplasmic state (Khakhlova and Bock, 2006). This may eliminate the insertion and/or deletions (indels) and the desire edited plastome will not be obtained.

Because of the presence of efficient homologous recombination in chloroplasts, genes in the chloroplast genome can be disrupted easily by inserting or replacing them with a selectable marker gene cassette. More subtle changes, such as induction of point mutations and small indels can be achieved by co-transformation of the selectable marker gene and a target gene with a specific mutation (Krech et al., 2013). Various chloroplast genes have been characterized by knockout mutations (Scharff and Bock, 2014). Variations in the selectable markers and their recycling techniques have made it possible to construct double and triple knockout mutants and the induction of mutations in multiple chloroplast genes, thereby allowing the discovery of various molecular interactions between the components of the photosynthetic complex (Fleischmann et al., 2011).

### Improving Photosynthesis Through Chloroplast Engineering

**FIGURE 5:**
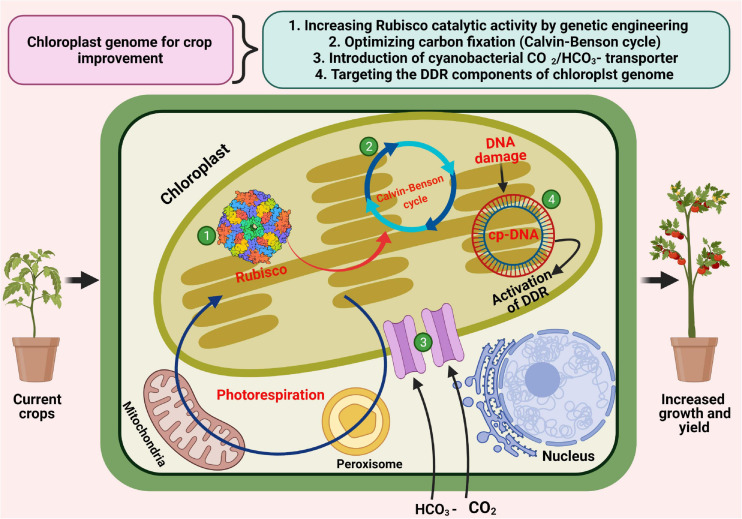
Modulation of chloroplast genome for crop improvement. Various aspects of employment of chloroplast genome for improving photosynthesis and crop productivity including modifications of Rubisco subunits through plastome engineering, optimization of carbon fixation, the introduction of cyanobacterial HCO_3_– and CO_2_ transporters, and targeting various components of DNA damage response (DDR).

Ribulose-1,5-bisphosphate carboxylase/oxygenase, also known as Rubisco is the most abundant class of protein in nature and appeared to be the major enzyme of the Calvin–Benson cycle, which is responsible for fixing atmospheric CO_2_ into the C_3_ organic acid 3−phosphoglycerate (3−PGA) for its further utilization to regenerate the substrate ribulose−1,5−bisphosphate (RuBP) or form the building blocks of carbohydrate synthesis (Sharwood, 2017). Rubisco is a large protein complex consisting of eight large catalytic subunits (rbcL) and eight small catalytic subunits (rbcS). The large subunits are encoded by the chloroplast genome itself, whereas, the small subunits are encoded by the nuclear genome, respectively. Rubisco has been shown to exhibit catalytic inefficiencies, including slow catalysis and imperfect discrimination between CO_2_ and O_2_, thus considered as the most obvious target for crop improvement to enhance the photosynthetic capacity of plants (Figure 5; Parry et al., 2013; Galmés et al., 2014; Pottier et al., 2018). The distinct locations of the genes responsible for encoding the Rubisco large (chloroplast) and small (nucleus) subunits have made the engineering of Rubisco complicated in higher plants. In this context, development of chloroplast transformation was the first breakthrough in engineering the Rubisco large subunit (Sharwood, 2017).

Among some of the initial trials of Rubisco large subunit engineering, the substitution of tobacco rbcL was achieved with the sunflower and cyanobacterial counterparts (Kanevski et al., 1999). Although, the resulting hybrid Rubisco was catalytically inactive or inefficient, this initial study provided proof-of-concept for this new technology. Another approach was to assemble the functional Rubisco enzyme within the chloroplast. To achieve this, Rubisco small subunit gene (rbcS) was relocated back into its pre-endosymbiotic location within the plastome (Whitney and Andrews, 2001; Zhang et al., 2002; Dhingra et al., 2004). One very recent study has demonstrated the effectiveness of various sequences on Rubisco protein production from synthetic rbcL-rbcS operons transformed into the chloroplast genome to replace rbcM. For this, a tobacco bRrDs line was generated, in which endogenous *rbcS* genes were silenced and the rbcL gene of tobacco was replaced using the *rbcM* gene of *Rhodospirillum rubrum* (Avila et al., 2020). The *rbcM* gene of *Rhodospirillum rubrum* has been found to encode the L2 form of Rubisco that does not assemble with the small subunit. Although the production of Rubisco was achieved only up to 50% of the wild type, this research has explored the possibilities of modulating different Rubisco subunit assemblies for increasing the production of Rubisco by chloroplast engineering. Apart from Rubisco engineering, another interesting strategy to enhance photosynthesis includes the introduction of a CO_2_-concentrating mechanism from cyanobacteria into transplastomic plants to maximize their carbon fixation (Lin et al., 2014; Occhialini et al., 2016).

### Molecular Breeding Through Modifications of Mitochondrial Genome

**FIGURE 6:**
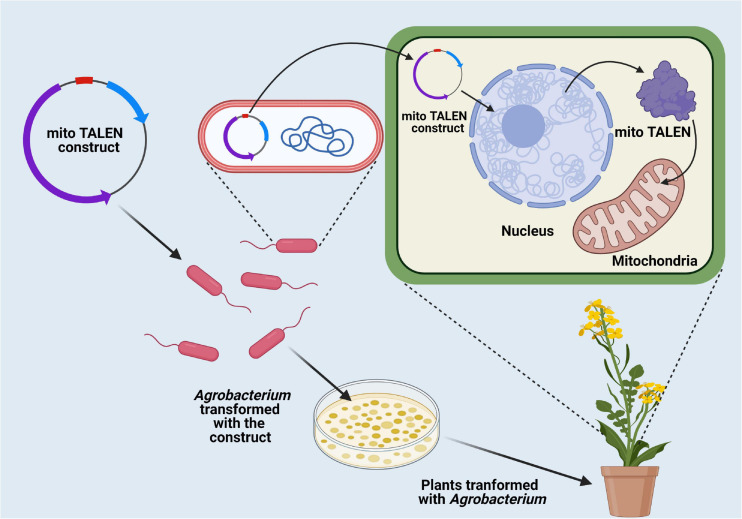
Transformation of the mitochondrial genome. Genome editing in mitochondria through transcription activator-like editing nucleases (TALEN) mediated nuclear transformation and generated new types of crop plants with improved productivity.

Although, the structure of angiosperm mitochondrial genome has been studied in detail, molecular analysis of mitochondrial genome for crop improvement has not been successful because of the lack of efficient transformation strategies. Genome editing in mitochondria has been made possible by employing transcription activator-like editing nucleases (TALEN)- mediated nuclear transformation, which resulted in new transformed rice and canola (Figure 6; Kazama et al., 2019). Generally, TALENs consist of two domains, including the DNA binding domain, which can be engineered to recognize any DNA sequences, and the nuclease domain, capable of nicking the DNA and causing deletions. For manipulating mitochondrial genes, the plant-adapted TALEN was modified with the inclusion of a mitochondrial localization signal sequence and the DNA binding domain was engineered in a way so that it can recognize the target genes of interest. The plasmid encoding the mito-TALEN was then transferred into plants by *Agrobacterium-*mediated transformation. In the above-mentioned study, two mito-TALENs were constructed, which targeted two mitochondrial genes, including *orf79* (rice) and *orf125* (rapeseed), respectively (Kazama et al., 2019). The resulting deletions have established role of these genes in male sterility, which appeared to prevent self-fertilization in certain hermaphroditic plants. Another recent study has demonstrated utilization of mito-TALENs to knock out the function of two mitochondrial genes, including *atp6-1* and *atp6-2*, respectively in the model plant *Arabidopsis thaliana* (Arimura et al., 2020). In addition to TALEN-mediated plant mitochondrial genome editing, recent studies have reported development of a CRISPR-free technique for human mitochondrial genome base editing (Mok et al., 2020). This newly developed genome editing technique utilizes a double-stranded DNA-specific interbacterial cytidine deaminase toxin to induce targeted mutation in the human mitochondrial genome with approximately 5-50% efficiency (Mok et al., 2020). Although, additional research is required to further decipher the efficiency of this approach.

### Conclusion and Prospects

Since their discovery about half a century ago, significant progress has been made on the sequencing and characterization of mitochondrial and chloroplast genome. Maintenance of organellar genome stability in the context of permanent threat from oxidative damage is one of the major challenges for eukaryotic cells. Despite this, it has long been considered that bona fide DNA damage repair mechanism is absent in both plant mitochondria and chloroplast genomes. However, recent advancements on the structure-function aspects of organellar genome have demonstrated the existence of highly efficient DNA damage repair machinery in both chloroplast and mitochondria and has become an emerging field of research within a little more than a decade for better understanding plant cell function under stress conditions. Previous studies have indicated the functioning of base excision repair and homologous recombination repair pathways for repairing oxidative damages and double-strand breaks, respectively in the organellar genomes. Besides its role in repairing DSBs, the HR pathway appears to be involved in organellar genome replication and maintenance of low mutation rates. However, corresponding knowledge on the mechanistic details of HR mediated functions remains still elusive and deserves further extensive research. In addition, there are still potential questions regarding the evolution and variation of plant organellar genomes and how the various DNA repair mechanisms in organellar genomes became closely associated during their evolution. These aspects needs further research to get more insight into the evolutionary aspects DNA damage response and repair mechanisms in the context of genome stability mechanisms in plants.

After the origin of mitochondria and chloroplast following the engulfment of ancestral α-proteobacteria and cyanobacteria, most genes were subsequently either lost or transferred to the nucleus through horizontal gene transfer after the endosymbiosis, and thus both organelles are dependent on nuclear-encoded proteins for their DNA damage response and genome stability maintenance. As organelles are critical integrators of both internal and external cues, various activities in the organelles are needed to be tightly coordinated with nuclear activities to enable plant development and stress signaling. Various crucial proteins for organelle genome maintenance, such as the WHIRLY family proteins are found to be under coordinated control of organelle and nuclear genomes. Dual localization of proteins associated with various important cellular responses in eukaryotic organisms has eventually been accepted as an important phenomenon linked with multi-functionalization and also proposed to have an evolutionary advantage.

To meet the demand for food for a rapidly growing world population in the face of an unsure global climate change and reduced arable land, improvement in crop production is emerging as an immediate priority. In this context, along with nuclear transformation, plants also offer the opportunity to transform the comparably smaller genomes of two semi-autonomous organelles, including chloroplast and mitochondria. The chloroplast genome has been modified genetically for improvement of crop yield, nutritional quality, and resistance to various abiotic and biotic stresses. However, mitochondrial transformation in higher plants was not been successful initially, but stepwise progress has been made for manipulation mitochondrial genes and their transcripts. In addition, emphasis has also been given to target various DNA damage responses and repair components associated with organellar genome maintenance for crop improvement. However, further research is required in this exciting field of research to unveil plant cell response under changing environment.

## Author Contributions

SR, KM, and SB researched and developed the idea. SR, KM, SB, SD, MM, and PR wrote the review. KM, SB, and SD created the figures. SR and KM edited the manuscript. All authors have read and approved the manuscript.

## Conflict of Interest

The authors declare that the research was conducted in the absence of any commercial or financial relationships that could be construed as a potential conflict of interest.

## Publisher’s Note

All claims expressed in this article are solely those of the authors and do not necessarily represent those of their affiliated organizations, or those of the publisher, the editors and the reviewers. Any product that may be evaluated in this article, or claim that may be made by its manufacturer, is not guaranteed or endorsed by the publisher.
